# The Use of Artificial Intelligence for Skin Disease Diagnosis in Primary Care Settings: A Systematic Review

**DOI:** 10.3390/healthcare12121192

**Published:** 2024-06-13

**Authors:** Anna Escalé-Besa, Josep Vidal-Alaball, Queralt Miró Catalina, Victor Hugo Garcia Gracia, Francesc X. Marin-Gomez, Aïna Fuster-Casanovas

**Affiliations:** 1 Centre d’Atenció Primària Navàs-Balsareny, Institut Català de la Salut, 08670 Navàs, Spain; aescale.cc.ics@gencat.cat; 2 Health Promotion in Rural Areas Research Group, Gerència d’Atenció Primària i a la Comunitat de la Catalunya Central, Institut Català de la Salut, 08242 Manresa, Spain; qmiro.cc.ics@gencat.cat (Q.M.C.); xmarin.cc.ics@gencat.cat (F.X.M.-G.); 3 Faculty of Medicine, University of Vic-Central University of Catalonia, 08500 Vic, Spain; 4 Unitat de Suport a la Recerca de la Catalunya Central, Fundació Institut Universitari per a la Recerca a l’Atenció Primària de Salut Jordi Gol i Gurina, 082424 Manresa, Spain; afuster.cc.ics@gencat.cat; 5 Servei d’Atenció Primària Osona, Gerència Territorial de la Catalunya Central, Institut Català de La Salut, 08500 Vic, Spain; 6 eHealth Lab Research Group, School of Health Sciences and eHealth Centre, Universitat Oberta de Catalunya (UOC), 08018 Barcelona, Spain

**Keywords:** artificial intelligence, primary health care, dermatology, skin diseases

## Abstract

The prevalence of dermatological conditions in primary care, coupled with challenges such as dermatologist shortages and rising consultation costs, highlights the need for innovative solutions. Artificial intelligence (AI) holds promise for improving the diagnostic analysis of skin lesion images, potentially enhancing patient care in primary settings. This systematic review following PRISMA guidelines examined primary studies (2012–2022) assessing AI algorithms’ diagnostic accuracy for skin diseases in primary care. Studies were screened for eligibility based on their availability in the English language and exclusion criteria, with risk of bias evaluated using QUADAS-2. PubMed, Scopus, and Web of Science were searched. Fifteen studies (2019–2022), primarily from Europe and the USA, focusing on diagnostic accuracy were included. Sensitivity ranged from 58% to 96.1%, with accuracies varying from 0.41 to 0.93. AI applications encompassed triage and diagnostic support across diverse skin conditions in primary care settings, involving both patients and primary care professionals. While AI demonstrates potential for enhancing the accuracy of skin disease diagnostics in primary care, further research is imperative to address study heterogeneity and ensure algorithm reliability across diverse populations. Future investigations should prioritise robust dataset development and consider representative patient samples. Overall, AI may improve dermatological diagnosis in primary care, but careful consideration of algorithm limitations and implementation strategies is required.

## 1. Introduction

Dermatological conditions pose a significant health concern that is frequently encountered in primary care settings. In the United States, individuals experience on average approximately 1.6 skin diseases per year, highlighting the prevalence and impact of these conditions [1,2]. Notably, dermatological issues account for approximately 20% of all visits to general practitioners (GPs), underscoring their significance as a common reason for seeking medical attention in primary care [1,2]. Skin disease ranks among the primary factors driving patients to seek medical attention from their GPs [3]. However, there is a persistent shortage of dermatologists, particularly in rural areas, and consultation costs are rising. The estimated direct healthcare cost of skin disease in the USA is USD 75 billion, including USD 46 billion in medical costs (office visits, procedures, and tests) and an additional USD 11 billion in indirect opportunity costs due to missed work or reduced productivity for patients and their caregivers [1].

However, the diagnostic accuracy of non-dermatology specialists varies, reportedly ranging from 24% to 70% [4,5]. A 1998 study in Spain, involving 3164 patients, examined the diagnostic concordance between primary care physicians and dermatology specialists and found a concordance of 65.52% [6]. On the contrary, in another study, the diagnostic agreement was 27.3%, showing the great variability that exists in the diagnostic accuracy of dermatological diseases [7].

Due to the diagnostic variability of non-dermatologist clinicians, improving the diagnostic accuracy of non-referred cases while reducing unnecessary referrals has enormous implications for healthcare systems [8]. Therefore, the appropriate diagnosis of dermatological conditions at the point of care in primary care could potentially lead to earlier diagnosis and treatment of any skin cancer and other skin diseases, thereby improving patient outcomes and satisfaction and increasing the capacity of dermatology practices [9,10].

Several studies have shown that artificial intelligence (AI) can have a positive impact on the diagnostic accuracy of healthcare professionals, significantly increasing concordance with the reference standard. Hekler et al. showed that the combination of human and artificial intelligence was superior to the individual results of dermatologists or deep neural networks in isolation [11]. Jain et al. described an increase in diagnostic agreement of 10% (95% CI, 8–11%; *p* < 0.001), from 48% to 58%, for primary care physicians (PCPs); for nurse practitioners (NPs), the improvement was 12% (95% CI, 10–14%; *p* < 0.001), from 46% to 58% [10].

AI is computer science that involves creating sequences of data-related instructions that aim to reproduce human cognition [12]. There are four main areas of AI that are applicable to medicine: machine learning, artificial neural networks, natural language processing, and computer vision. Since a fundamental part of dermatology is the assessment of skin lesions, many AI studies focus on machine learning and artificial neural network applications for image classification to improve the accuracy of skin disease diagnostics [13]. AI can lead to more accurate dermatological diagnoses through automated segmentation analysis of clinical, dermoscopic, and even histopathological images [14]. Dermoscopy is a non-invasive diagnostic tool for skin lesions, including skin cancer. It is performed using a hand-held dermatoscope that uses a transilluminating light source to magnify skin lesions and allow for the visualisation of subcutaneous skin structures within the epidermis, dermoepidermal junction, and papillary dermis [15]. Dermoscopy has been shown to improve the accuracy of dermatologists in diagnosing malignant melanoma when compared to clinical assessment with the naked eye alone. Dermoscopy is becoming increasingly useful in primary care, improving practitioners’ sensitivity for skin cancer [16,17].

These developments have led computer scientists to apply these techniques to develop algorithms capable of recognising some of these skin lesion images, particularly skin cancer. AI models can perform binary classification based on clinical images to distinguish between benign and malignant skin lesions. For example, they can distinguish keratinocyte carcinoma from seborrheic keratosis and nevus from melanoma with a level of accuracy comparable to that of dermatologists [8,14,18,19].

**Figure 1:**
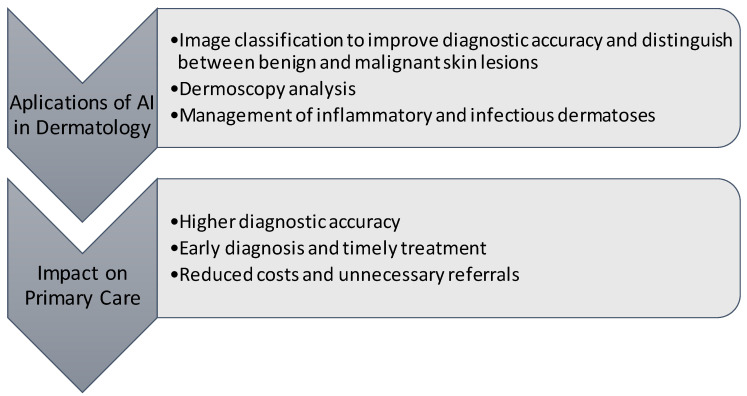
Diagram of potential use cases of AI in dermatology.

Furthermore, in recent years, the use of neural networks has improved the management of other skin conditions, such as inflammatory dermatoses, infectious lesions, and the detection of cutaneous manifestations of COVID-19 [20,21,22,23,24]. These conditions may be more difficult to classify due to greater clinical heterogeneity (i.e., atopic dermatitis), similar clinical presentation (i.e., acne vs. rosacea, psoriasis vs. eczema, and cutaneous T-cell lymphoma vs. eczema), numerous subtypes (i.e., psoriasis), or greater variance in severity, and will likely require more complex algorithms to grade disease severity and generate accurate differential diagnoses [25]. See a schematic diagram of the main potential use cases in Figure 1.

To assess the applicability of AI in clinical practice, it is important that the algorithms are trained on representative databases of all pathologies, ethnicities, skin colours, genders, etc., as well as on sufficient clinical and dermoscopic images to improve the reliability of the results. Most AI models are trained on theoretical images from image datasets. There have been few studies performed in routine clinical practice settings employing non-standardised imaging, so the prospective validation of these tools in real life is imperative. In Europe, the current governing regulation is the Medical Device Regulation (Regulation 2017/745), which has been in force since May 2020 and repeals Directive 93/42 [26,27]. In the case of the application of machine learning (ML) models as complementary diagnostic tools, different groups of experts around the world have developed guidelines to stipulate the essential requirements to be assessed in this practice to confirm that the application of these algorithms in clinical practice works and to evaluate their potential impact [28,29]. In the USA, the International Medical Device Regulators Forum (IMDRF), in collaboration with the FDA, has developed a standardised framework for medical devices. This framework includes standardising definitions, categorising risks, managing quality, and evaluating clinical aspects, aiming to balance innovation and patient safety in the fast-evolving field of medical software [30]. In this context, no medical devices using generative AI were approved by the FDA until October 2023. At that time, the FDA listed 691 approved medical devices with artificial intelligence, of which 108 were approved in 2023 [31].

As mentioned above, PC is where most consultations for skin conditions are received, but there have been few studies conducted in this setting. Some studies have included PC GPs alongside dermatologists as image readers to compare the performance of the models with that of the specialists [32]. Other studies have concluded that AI tools could be used in PC, resulting in a new tool for diagnostic support, screening and expanding differential diagnosis by non-experts [9,32]. However, this has not been widely studied and the evidence is insufficient. Therefore, there is every reason to believe that AI tools could improve the diagnostic accuracy and diagnostic satisfaction of professionals and patients in primary care (PC) settings.

The aim of this study was to systematically review AI techniques that can be used to diagnose skin disease in PC. We deliberately focused this review on the applicability of diagnostic algorithms to primary care, where the prevalence of consultations for skin problems is high and where GPs have a lower diagnostic accuracy than specialist clinics. This is also the setting where AI models may have the greatest benefit, as this is where the initial assessment of most suspicious skin lesions takes place. We analysed the quality of the evidence, the usefulness of the algorithms, the different types of skin conditions for which AI is used, the impact on primary care, and the potential for use in PC.

## 2. Materials and Methods

This systematic review was conducted in accordance with the Preferred Reporting Items for Systematic Reviews and Meta-Analysis (PRISMA) statement [33], and the protocol was registered with PROSPERO CRD42023403395 before this review was conducted.

### 2.1. Search Criteria

A comprehensive search strategy was developed to identify articles reporting AI techniques that can be used to diagnose skin disease in primary care. An assortment of terms related to these main concepts were used: [“Artificial intelligence”] OR [“computer-assisted diagnosis”] OR [“machine learning”], AND [“Primary health care”] AND [“Dermatology”] (see detailed search criteria in the Appendix A). Database searches were conducted in the PubMed, Scopus, and Web of Science bibliographic databases, using keywords related to AI, skin diseases, and primary care.

Manual searches of bibliographies, citations, and related articles (PubMed function) of included studies were performed to identify any additional relevant articles that may have been missed by the searches.

### 2.2. Inclusion and Exclusion Criteria

All primary research articles published in English in peer-reviewed journals between 1 January 2012 and 31 December 2022 were included if they met the inclusion criteria. The timeframe starting 1 January 2012 was chosen based on a review of the existing literature, which indicated that the majority of studies focused on this 10-year period. This is probably due to the notable advancement in machine learning performance following the emergence of deep learning around that time [34]. It was also during that year that a deep learning model won the ImageNet object recognition challenge and outperformed competing approaches by a significant margin, which was notable progress in automatic visual recognition [35].

We included studies that described the diagnostic performance of AI models in the context of PC and their relationship to skin diseases. Additionally, we considered studies that compared the performance of AI models with that of general practitioners (GPs) specifically in the field of medical imaging for skin diseases. Studies that only described the development of an AI algorithm without undertaking any testing or evaluation or that did not include an element of AI were excluded. Manuscripts were excluded if they were narratives, editorials conference papers, case studies, surveys, book chapters, did not apply to primary care, or the full text was not available in English.

### 2.3. Study Selection

Covidence was used to remove duplicate results and facilitate the screening process [36]. A.E.-B. and V.G. conducted the initial screening of titles and abstracts after duplicate removal to identify studies meeting inclusion criteria. J.V.-A. resolved any initial disagreements during title and abstract screening. A.E.-B. and VG independently conducted the full-text screening of each document for the eligibility criteria, which were as follows: (1) document was a research study, (2) used AI-assisted tools in clinical practice for the diagnosis of skin disease, and (3) used primary care data and/or was conducted in a primary care setting and/or explicitly demonstrated the applicability of the study to primary care. J.V.-A. resolved any disagreements during full-text screening. A.E.-B. and V.G. independently performed data extraction using a pre-designed data extraction sheet. J.V.-A. resolved any disagreements in the extracted data.

The primary outcome considered the diagnostic accuracy of AI algorithms in skin diseases. The secondary outcomes considered AI/ML algorithm design, the appropriateness of the datasets used to develop the AI algorithm, the usefulness of AI in the management of skin diseases, the primary care implication of the studies, and quality assessment. Based on the information extracted from each study, the following categories were highlighted: publication details, characteristics of the included studies (country, design, and gold standard), participant characteristics (demographics and skin type), AI tool characteristics (AI algorithm method, dataset characteristics, image type, skin diseases included, and usefulness), primary care function(s) in the study, and study performance outcomes, separating the studies whose outcomes are algorithm metrics from those where the outcome is the variation between GP diagnostic accuracy and that of AI. An “unknown” category was used if not enough information was provided for category selection.

The studies identified were heterogeneous, using different AI techniques and evaluating algorithms in different ways using different outcome measures. For this reason, a meta-analysis was not considered appropriate; instead, we used a narrative synthesis approach, following established guidelines for the methodology of this review.

**Figure 2:**
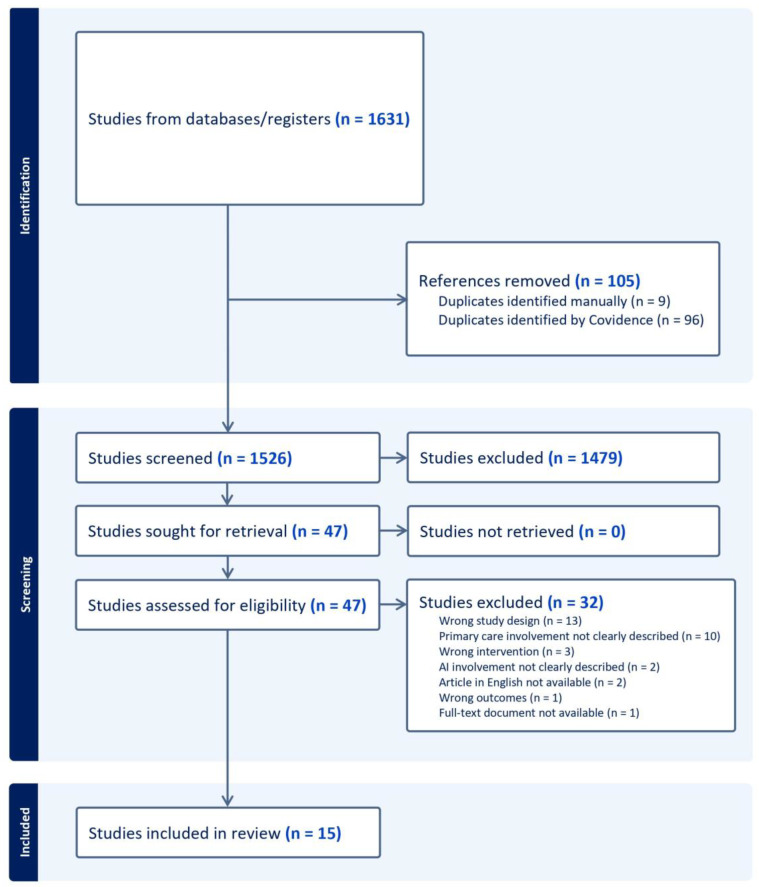
PRISMA flowchart of included and excluded studies in a systematic review.

The search yielded 1526 non-duplicate documents for title and abstract screening; 47 met the eligibility criteria for full-text screening and 15 met the final criteria as shown in Figure 2 (PRISMA flow diagram). See the PRISMA flowchart in Figure 2.

Of the thirty-two excluded manuscripts, thirteen were excluded as they did not meet the study design criterion, most of these because they were conference papers or reviews with no accuracy results, and four of these had a wrong or undescribed intervention or outcome. Ten were not related to primary care or it was not clearly described; two of them were excluded due to being written in a language different from English; one full text was not available; and two articles used a software which was not AI.

### 2.4. Risk-of-Bias Assessment

Risk of bias was independently assessed by A.E.-B. and V.G. for all included studies using the standard QUADAS-2 [37] critical appraisal tool [38]. An overall assessment of whether each paper had a high, low, or unclear risk of bias in the classic domains (patient selection; index test; reference standard; and flow and timing) was included. The QUADAS-AI critical appraisal tool [39] was used, adding some domains to assess more specific elements of quality assurance for the use of AI in medicine. Applicability to real clinical practice, diversity of patient selection, generalisability of the algorithm and external evaluation were assessed. Any disagreements in the assessment were resolved by a third reviewer (J.V.-A.).

**Figure 3:**
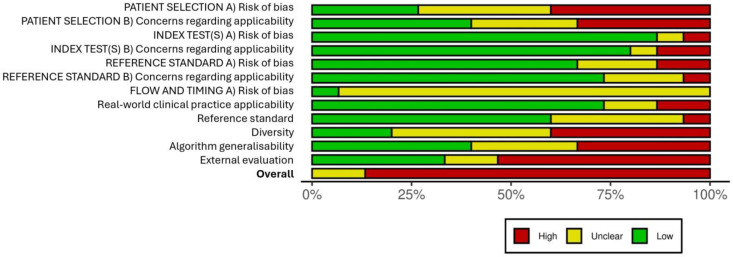
Quality assessment plot.

The results of the quality assessment for each study in each domain can be found in the Supplementary Materials (Figure S1) while the overall mean risk of bias for each domain can be consulted in Figure 3.

The risk of bias in the patient selection domain was generally high, as many studies did not take into account the timing, randomisation process, or inclusion and exclusion criteria while selecting the participants. In the “index test” domain, the risk of bias was generally low, as the development of CNNs was adequately described in most studies, and optimal databases were used for training and testing the algorithms in terms of size and homogeneity. The reference standard also showed a low risk of bias, as a significant number of the studies clearly detailed the interpretation characteristics of the reference standard’s results, and no biases were suspected to have been introduced.

Regarding the “flow and timing” domain, the available information provided by the studies on the interval between the reference standard and the index test, the number of patients who received the tests, and on whether those patients received the exact same test was limited, resulting in an “unclear” outcome.

The risk of bias regarding the applicability of AI in clinical practice was assessed as “low” since the algorithms generally outperformed the reference standard in the centres where the study was conducted, demonstrating their potential usefulness.

Regarding the risk of bias in the diversity domain, it is important to note that the samples used were relatively heterogeneous considering the number of patients included, which could introduce biases and not accurately represent the characteristics of primary care patients. Only eight out of the fifteen included studies describe the participants’ skin type. Three of them include Fitzpatrick subtypes I–IV, while the remaining five include all Fitzpatrick subtypes. However, the representation of subtypes V and VI was quite low, which constitutes a significant limitation in the applicability of the algorithms for patients with dark skin.

Despite the results obtained by each algorithm in their analysis, they were generally tested in a limited number of healthcare centres, which could introduce biases when using these algorithms in different settings with different demographic characteristics, casting doubt on their generalisability.

Finally, it is important to acknowledge that several studies included in the review may have an increased risk of bias due to conducting external validation tests of the algorithms using databases that may not be suitable for this purpose.

## 3. Results

### 3.1. Search Outcomes

There were 1631 articles retrieved in the literature search. After removing duplicates, a screening of 1526 titles and abstracts was conducted against the inclusion and exclusion criteria. Of these, 1479 articles were excluded, and 47 were subjected to a full-text review. Ultimately, 15 studies met the final selection criteria and were included in this review (Figure 2).

### 3.2. Study Characteristics

The full study characteristics are shown in Table 1 and Table 2. As described in Table 1, 15 studies were included in this review (full list of included studies is included in Supplementary Materials, with publication dates ranging from 2019 to 2022. Most of these studies were conducted in Europe (*n* = 5) and the United States (*n* = 4), while two were conducted in Brazil, one in Argentina, one in Chile, one in India, and one in East Asia. Three of the European studies also included work in Australia, and one of these included work in New Zealand.

A total of 14/15 studies included were diagnostic accuracy studies, and only 1/15 was an image-based retrospective study. Philips et al. [39] also carried out a meta-analysis following the diagnostic accuracy study to compare the accuracy of their algorithm with that of current diagnostic practice.

Regarding the gold standard chosen by each study, 2/15 used histopathology alone, 6/15 used dermatologist consensus alone, 4/15 combined histopathology and dermatologist consensus, 1/15 combined histopathology and dermatological surveillance, 1/15 combined face-to-face clinic visits and dermatologist consensus, and 1/15 study did not describe its gold standard.

As described in Table 2, there was considerable variation in the number of participants included in each study. The authors considered that this variable should be included as it is of general interest in comparing the different studies, but it is important to note, to avoid confusion, that it is described as the number of patients or images used to test the algorithms specifically for this study, not for development, which is described later in the characteristics and distribution of the datasets.

The number of participants ranged from 16,114 in the largest study to 100 in the smallest. In the study by Liu et al. [22], the number was significantly higher than in the other studies, as it was a study on the development of an algorithm with a large database of images corresponding to this large number of cases (*n* = 16,114). It was the same algorithm used in subsequent studies, such as the same study included in the systematic review by Jain et al. [10]. Two of the fifteen studies do not report the exact number of participants. The first part of the study by Phillips et al. [39] evaluated the diagnostic accuracy of an AI algorithm. However, the second part of the study was a meta-analysis to compare the diagnostic accuracy of this algorithm with that of other AI algorithms. However, based on the assessment of 32,226 pigmented lesions, the authors did not consider them all to be participants in the study, as they were the sum of the 82 studies included in the meta-analysis, so they left this row blank, but included the study in the systematic review.

Giavina-Blanchi et al. conducted an AI algorithm development study using clinical and dermoscopic images, so the data were described under algorithm test development [42].

In the study by Lucius et al. [44], 233 participants were divided into three datasets for three separate accuracy test experiments according to different characteristics. This number of participants was the sum of 163, 35, and 35 images, respectively.

With regard to the gender of the participants, 10/15 articles did not provide this information, while in 5/15 articles, the percentage of females varied from 65.2% to 45.4%. A total of 9/15 articles did not provide information on the age of the participants. In the studies that did mention age, 4/15 included patients aged between 18 and 65 years, 1/15 included patients aged between 58 and 78 years, and 1/15 included only people aged over 18 years.

Nine of the fifteen studies did not report the ethnicity of the participants. Three studies included participants of multiple ethnicities, one study included only the Caucasian ethnicity, one study included the South Asian ethnicity, and one study included East Asian ethnicity.

As for the Fitzpatrick skin type scale, 5/15 studies included all subtypes, 2/15 included Fitzpatrick types I–IV, 1/15 included Fitzpatrick types I–II, and 7/15 studies did not include any subtypes.

### 3.3. Primary Outcome

#### Diagnostic Accuracy of AI Algorithms in Skin Diseases

The main objective of this study was to evaluate the performance accuracy results of the different AI models. The results were divided into two main groups, depending on whether they extracted the results from the model (Table 3) or compared the increase in diagnostic agreement with the gold standard with or without the use of the AI tool (Table 4).

In 14/15 studies, values for the AI model’s diagnostic metrics were available, and 4/15 of these included a comparison of the clinician’s diagnostic agreement rate with the gold standards when assisted by AI versus unassisted.

Table 3 shows the 14 studies in which metrics could be compared to assess the diagnostic accuracy of each algorithm. The results were heterogeneous, with some studies categorised according to different models evaluated (Giavina-Bianchi, M et al. [43], Tschandal, P et al. [32]), according to the top one or top three diagnoses (Dulmage et al. [41], Liu, Y et al. [22], Muñoz-López, C et al. [45]), according to type of skin disease with a binary classification (Thomsen, K et al. [25]), and according to the quality of image and/or clinical data acquisition (Lucius, M et al. [44]).

The most reported parameters were accuracy (11 of 14), sensitivity (10 of 14), and specificity (9 of 14).

The sensitivity of the different studies sits between 0.58 and 0.96 and the accuracy between 0.41 and 0.93. In both cases, the lowest parameter corresponds to the top one category of the algorithms, which showed the skin diagnoses as a list, and improved significantly when they took into account the first three (top three) diagnoses.

Some studies, specifically four, aimed at assessing the difference in clinicians’ diagnostic agreement when using the AI model or not (Table 4). In all of them, AI assistance was significantly associated with higher agreement with reference diagnoses. The relative differences in diagnostic agreement were represented with the percentage of increase in the diagnostic accuracy of primary care professionals (GP or nurse practitioners) or in AUC values.

The largest difference was seen in the study by Dulmage, B. et al. [41], where the use of AI tool support was associated with a 32% increase in diagnostic accuracy in defining the morphology of the primary lesion. The study carried out by Jain, A et al. [10] showed that the increase in diagnostic agreement for PCPs was 10% (95% CI, 8–11%; *p* < 0.001), from 48% to 58%; for NPs, the increase was 12% (95% CI, 10–14%; *p* < 0.001), from 46% to 58%. They also showed that AI support was significantly associated with higher agreement with the biopsy-obtained diagnosis categories of malignant, precancerous, or benign, which increased by 3% (95% CI, −1% to 7%) from 64% to 67% for PCPs and by 8% (95% CI, 3% to 13%) from 60% to 68% for NPs. Rates of biopsy request decreased, and diagnostic concordance increased in cases not indicated for referral to dermatology.

Lucius, M. et al. [44] showed that when GPs were given the opportunity to access the output of EffcientNetB5 per image, global accuracy increased to 42.42%. This was an increase of 25.13%, representing an improved ability to classify pigmented skin lesions, particularly basal-cell carcinoma and melanoma.

Zhang, Yu et al. [49] showed that two general practitioners significantly improved their diagnostic performance, with AUC values increasing from 0.537 and 0.575 to 0.778 and 0.788, respectively.

### 3.4. Secondary Outcomes

#### 3.4.1. AI/ML Algorithm Design

The most common AI techniques used in the studies included in this systematic review are artificial neural networks, specifically convolutional neural networks (CNNs), in 8/15 of the studies, and deep neural networks (DNNs), in 6/15 of the studies. In one of the studies, the machine learning type used was not clearly described.

#### 3.4.2. Appropriateness of Datasets Used to Develop the AI Algorithm

Among the available data, the vast majority were large databases that had been used for training, with a distribution between 10 and 20% in the test set and validation set in most cases. External validation of the algorithm was only available for five studies. Two of them relied solely on the external validation of algorithms [47,50].

The number of images included in the training set was between 1088 and 220,680.

Some studies (8/15) did not report the total dataset distribution on the methods of the algorithm development, and in these cases, the authors could not determine the distribution of the datasets. Phillips, M. et al. [39] only reported the total sample used to train the algorithm. In addition, Jain et al. [10] used the AI tool described by Liu et al. (Table 5).

#### 3.4.3. Usefulness of AI in the Management of Skin Diseases

As detailed in the Table S1 in Supplementary Materials, 5/15 studies included in this review used AI for the triage of suspicious skin lesions, 7/15 used algorithms as diagnostic support tools, and 3/15 used algorithms to serve both functions.

In all cases, modality triage tools refer to models trained on tumoral images. In contrast, tools that aim to provide a nominal diagnosis of the skin lesion itself cover a wider range of lesions, including inflammatory and infectious pathologies in addition to tumour pathologies.

#### 3.4.4. Types of Skin Diseases in which AI Is Used

**Figure 4:**
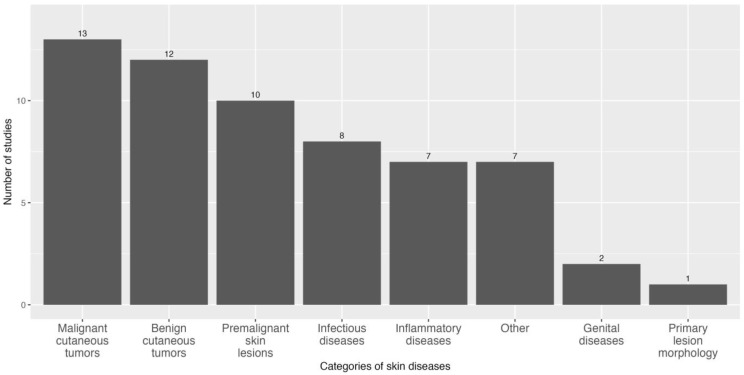
Types of skin diseases included in the studies.

Figure 4 shows the categories of skin diseases included, and the Table S1 shows the details of each skin lesion for each category. Thirteen of the fifteen studies included in this systematic review included tumoral lesions. In the benign category (12/15), the most common lesions were melanocytic nevus, dermatofibromas, seborrheic keratosis, and lentigo. In the premalignant category (10/15), the lesion described was actinic keratosis. As for malignant cutaneous tumours (13/15), the majority of these were melanoma, basal-cell carcinoma, and squamous-cell carcinoma.

Inflammatory or infectious lesions were increasingly included as the diagnostic target of AI algorithms and were included in eight and seven studies, respectively.

In terms of inflammatory pathologies, the studies mainly evaluated psoriasis, seborrheic dermatitis, eczema, urticaria, and hidradenitis, among a wide range of pathologies included. Among the infectious pathologies evaluated in the studies, we found that the main ones were acne, rosacea, tinea, pytiriasis versicolor, and warts, among others.

Many studies (7/15) also included diseases that did not fit into any of the categories mentioned earlier and these are grouped under the category of “others”. These include conditions such as vitiligo, androgenetic alopecia, alopecia areata, scars, etc. The aim of one of the studies included in this review (Dulmage, Brittany et al. [41]) was not to diagnose the pathologies themselves, but rather to diagnose the primary lesions.

Most of the studies did not differentiate between the different parts of the body where the lesions were located. However, one exception was the study by Yu, Z. et al. [49], which focused exclusively on lesions located on the scalp.

#### 3.4.5. Primary Care Implication of the Studies

**Figure 5:**
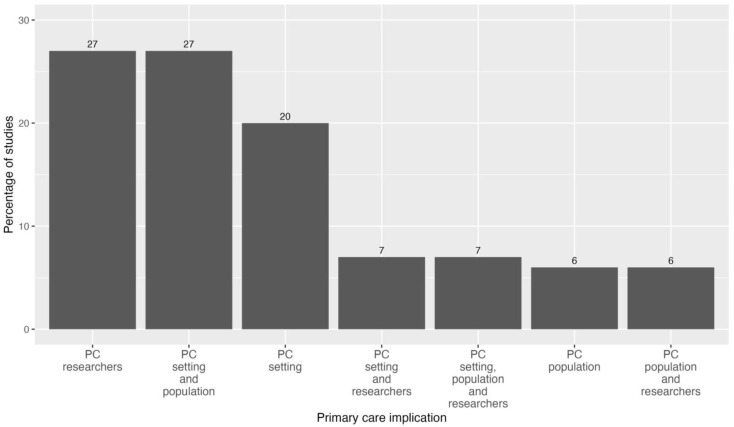
Primary care implication.

Figure 5 shows the primary care implication of the studies. One of the inclusion criteria for this systematic review was that the studies had to be specifically related to primary care. One of our main objectives was to examine the involvement of primary care in research related to AI in healthcare. It was observed that in nine out of the fifteen included studies (61%), PC was explicitly defined as the field of study or the applicability of the AI models (PC setting). Additionally, the primary care population served as the study subjects in 46% of the studies analysed (PC population). Finally, it is also common for primary care professionals to act as study researchers (47%), usually as readers of the skin images, comparing their diagnostic accuracy with that of the AI model (PC researchers).

Five of the fifteen studies included two of the three domains assessed for PC involvement, the most common combination (four studies, 27%) being studies that included subjects recruited from PC as the study population and also defined PC as the main study setting.

The study with the greatest involvement of primary care was the one carried out by Jain, A et al. [10]. In this study, PC was present in all three of the dimensions described. Twenty PC physicians and twenty nurses reviewed 1048 retrospective cases from a teledermatology practice service, including images that were referred from PC patients.

## 4. Discussion

This systematic review aims to examine the available scientific literature on the application of AI algorithms, primarily convolutional neural networks (CNNs), in the diagnosis of dermatological diseases in PC. There are numerous studies and reviews on the use of AI for skin disease diagnosis, but studies on its implementation in PC are limited. To the best of our knowledge, this is the first systematic review aimed at evaluating the use of AI across a wide range of skin lesion presentations, including tumours and inflammatory and infectious pathologies, in PC.

There are several studies, some of them similar to ours, that focus on tumoral pathologies and skin cancer [8].

We found a wide variation between the 15 studies included. Nevertheless, they are similar in terms of study design, as most of them are diagnostic accuracy studies, following, in most cases, the same development process, training, and internal validation of an AI algorithm with a dataset of clinical and/or dermatoscopic images, in most cases from a public database and in fewer cases from images collected retrospectively or prospectively in clinical practice. In terms of results, the most described main objective is diagnostic accuracy values (accuracy, sensitivity, and specificity), followed by evaluating the diagnostic accuracy of primary care professionals.

The heterogeneity of the included data is plausible given the description of the datasets used, the characteristics of the populations included, and the metrics used to express the results, among others, making it difficult to compare the different studies included here, which means that this systematic review was mainly descriptive.

In terms of the AI models used, all algorithms were developed using CNNs and DNNs, which are technically very similar deep learning techniques. Deep learning is the latest advancement in machine learning and is characterised by the fact that it learns directly from the input data without the need to extract human features. This is to be expected in the case of image analysis, as CNNs exhibit remarkable capability in feature extraction and thus have been widely used for image classification [34,51].

Regarding the impact of AI on the diagnosis of skin lesions in PC, it is interesting to highlight the four studies that aimed to evaluate an increase in diagnostic accuracy with or without the use of AI for the management of skin lesions in PC consultations. The diagnostic accuracy of PC professionals described in the four studies is consistent with that described in the literature [4,5] and demonstrates that the use of AI for the diagnosis of skin lesions in PC increases the diagnostic accuracy of professionals.

Notably, this review includes a large number of studies using clinical images that are or are not associated with dermoscopic images. Dermoscopy is a tool that is increasingly used in PC consultations and professionals are becoming more familiar with the technique, and its use increases diagnostic accuracy, especially in pigmented lesions [16,17]. Nevertheless, and following standard clinical practice, studies seem to be more representative of the PC setting when they have clinical images as the main study focus and dermoscopy as a complementary technique, as dermoscopy may not be available in some PC centres. One of the studies with the largest number of clinical images is that of Giavina-Bianchi et al. [42], with 140,446 clinical images obtained from a teledermatology service and distributed in different datasets to evaluate optimal DNN results.

Another fundamental aspect related to the viability of these algorithms in routine clinical practice is that the gold standard in all studies was aligned with clinical practice, with the consensus of dermatologists being the most commonly used reference parameter, together with lesion histopathology where available, predominantly in suspected malignant pathology.

In this review, model image analysis is mainly conducted as a binary classification of benign/malignant lesion or, in one of the studies, between two pathologies that may be difficult to differentiate clinically, such as acne and rosacea, psoriasis and eczema, or eczema and cutaneous T-cell lymphoma [25]. However, more and more algorithms lead to a list of three to five differential diagnoses, as the results show. This is very significant in PC consultations, where the clinician needs to have a rapid response, firstly to determine the malignant potential of the lesion and, if referral to dermatology consultation is required, to prioritise it for the appropriate use of resources and waiting lists. Secondly, it is important to provide a therapeutic and follow-up response, which is greatly aided by having a list of possible diagnoses for the lesion.

These results could serve as evidence that the implementation of AI algorithms in primary care would enhance the performance of healthcare systems in terms of diagnostic accuracy, reduction in unnecessary referrals and diagnostic tests (such as biopsies), alleviation of healthcare burden, and reduction in excessive resource expenditure, particularly at the primary care level.

### Strengths and Limitations

The main strength of our review is the rigorous methodology of the systematic review approach, despite our inability to conduct a meta-analysis due to the heterogeneity of the included studies. This review provides a comprehensive overview of the evidence for the use of AI in dermatology in primary care, an area that is often understudied. In addition, a complex quality assessment template was designed to evaluate every aspect that could introduce bias into the whole process of the development and the internal and external validation of the algorithms.

It was in this last point, in quality assessment, where we had the most difficulty in overcoming the limitations of adapting the standard QUADAS-2 quality analysis tool to AI algorithm studies. Firstly, our study involved an in-depth analysis of each domain, which was challenging due to the heterogeneity of the included articles. Additionally, we faced the task of adapting the standard QUADAS-2 quality analysis tool to suit AI algorithm studies by incorporating additional QUADAS-AI variables. Throughout the process, we placed particular emphasis on the domains that we believed were most relevant to the studies included in this systematic review [52].

Secondly, this review was limited by substantial variability in the quality of reporting and study designs. Some of the included datasets mainly consisted of lesions from patients recruited in specialised clinical settings, where the types and prevalence of cutaneous lesions might differ from those observed in primary care. Any diagnostic approach should be evaluated using data reflecting the patient population and disease prevalence in the intended environment; otherwise, diagnostic performance will be subject to spectrum bias. Furthermore, some of the datasets had small sample sizes and did not clearly separate training, testing, and validation datasets, leading to falsely high precision results due to overfitting.

Consistent with previous reviews [8,53,54], the main difficulty of comparing AI algorithms is the lack of complete information on the methods used to develop the models, the sometimes non-public datasets used, and the risk of bias and overfitting of the datasets. Above all, there is a notable scarcity of prospective studies and external validation to evaluate the performance of these algorithms beyond their training data. This refers to assessing how well the algorithms perform when presented with images that differ from those they were initially trained on.

## 5. Conclusions

This systematic review shows the potential of AI algorithms to improve diagnostic accuracy and agreement in the diagnosis of skin diseases in primary care. However, the heterogeneity of the studies and the limited information on certain aspects, such as participant characteristics and external validation, highlight the need for further research. Future studies should focus on diverse populations, develop appropriate datasets for algorithm validation, and consider the inclusion of more representative and diverse patient samples. Overall, AI has the potential to improve dermatological diagnosis in PC, but careful consideration of algorithm limitations and appropriate implementation strategies are required.

## Figures and Tables

**Table 1:** Study characteristics.

Authors	Country	Study Design	Publication Date	Gold Standard
Anderson, Jane et al. [40]	United States	Diagnostic test accuracy study	2022	Histopathology
Dulmage, Brittany et al. [41]	United States	Diagnostic test accuracy study	2020	Dermatologists’ consensus
Giavina-Bianchi, M. et al. [42]	Brazil	Diagnostic test accuracy study	2021	Dermatologists’ consensus
Giavina-Bianchi, M. et al. [43]	Brazil	Diagnostic test accuracy study	2021	Dermatologists’ consensus
Jain, A. et al. [10]	United States	Diagnostic test accuracy study	2021	Dermatologists’ consensus Histopathology
Liu, Y. et al. [22]	United States	Diagnostic test accuracy study	2020	Dermatologists’ consensus
Lucius, M. et al. [44]	Argentina	Diagnostic test accuracy study	2020	Not described
Muñoz-López, C. et al. [45]	Chile	Diagnostic test accuracy study	2020	In-person clinic visit Dermatologists’ consensus
Pangti, R. et al. [46]	India	Diagnostic test accuracy study	2020	Dermatologists’ consensus
Phillips, M. et al. [39]	Europe Australia	Diagnostic test accuracy study, meta-analysis	2019	Histopathology
Sangers, T. et al. [47]	Europe Australia New Zealand	Diagnostic test accuracy study	2022	Dermatologists’ consensus Histopathology
Soenksen, LR. et al. [48]	Europe	Diagnostic test accuracy study	2021	Dermatologists’ consensus
Thomsen, K. et al. [25]	Europe	Image-based retrospective study	2020	Dermatologists’ consensus Histopathology
Tschandl, P. et al. [32]	Europe Australia	Diagnostic test accuracy study	2019	Histopathology Dermatologists’ monitoring
Yu, Z. et al. [49]	East Asia	Diagnostic test accuracy study	2022	Histopathology Dermatologists’ consensus

**Table 2:** Participant characteristics [10,22,25,32,39,40,41,42,43,44,45,46,47,48,49].

Authors	Participants (n)	Sex (%)	Age Range	Ethnicity	Skin Type
Anderson, Jane et al.	100 *	Not disclosed	Not disclosed	Not disclosed	Not disclosed
Brittany Dulmage et al.	222 *	Not disclosed	Not disclosed	Not disclosed	All of them
Giavina-Bianchi, M. et al.	6945	Not disclosed	Not disclosed	Not disclosed	Not disclosed
Giavina-Bianchi, M. et al.		Not disclosed	>18	Not disclosed	Not disclosed
Jain, A. et al.	1016	64.2% female	18–65	Mixed ethnicity	All of them
		35.8% male			
Liu, Y. et al.	16,114	63.1% female	18–65	Mixed ethnicity	All of them
Lucius, M. et al.	233 (separated into three experiments 163 + 35 + 35)	Not disclosed	Not disclosed	Caucasian	All of them
Muñoz-López, C. et al.	281	63% female	18–65	Mixed ethnicity	Fitzpatrick I–II
		37% male			Fitzpatrick III–IV
Pangti, R. et al.	5014	Not disclosed	Not disclosed	South Asian	Not disclosed
Phillips, M. et al.		Not disclosed	Not disclosed	Not disclosed	Not disclosed
Sangers, T. et al.	372	50.8% female	58–78	Not disclosed	Fitzpatrick I–II
		49.2% male			Fitzpatrick III–IV
Soenksen, LR. et al.	133	Not disclosed	Not disclosed	Not disclosed	All of them
Thomsen, K. et al.	2342	Not disclosed	Not disclosed	Not disclosed	Fitzpatrick II–III
Tschandl, P. et al.	1511 *	Not disclosed	Not disclosed	Not disclosed	Not disclosed
Yu, Z. et al.	617	45.4% female	18–65	Asian/Middle Eastern	Not disclosed
		54.6% male			

* Number of images used, not patients.

**Table 3:** Outcome measures reported for individual models in the included studies [10,22,25,32,39,40,41,42,43,44,45,46,47,48,49].

Authors	Categories	SEN	SPE	Accuracy	AUC	PPV	PNV
Anderson, Jane et al.		0.80	0.95	0.92		0.80	0.95
Dulmage et al.	Top 1			0.68			
	Top 3			0.80			
Giavina-Bianchi, M. et al.		0.91	0.98	0.90			
Giavina-Bianchi, M. et al.	Dermoscopy model	0.90	0.89	0.89	0.96	0.64	0.98
Jain, A. et al.	Clinical model	0.91	0.84	0.85	0.94	0.57	0.98
Liu, Y. et al.	Top 1	0.58		0.71			
	Top 3	0.83		0.93			
Lucius, M. et al.	Low image resolution			0.76			
	High image resolution			0.78			
	Low-resolution images + clinical data			0.79			
	High-resolution images + clinical data			0.80			
Muñoz-López, C. et al.	Top 1			0.41			
	Top 3			0.64			
Pangti, R. et al.	Ext Top 1		0.99	0.75	0.9	0.61	0.99
	Ext Top 3			0.89			
	Int Top 1			0.77	0.95		
Phillips, M. et al.		0.85	0.85		0.93		
Sangers, T. et al.		0.87	0.70	0.76		0.61	0.91
Soenksen, LR. et al.		0.89	0.90	0.85	0.97		
Thomsen, K. et al.	Psoriasis vs. eczema	0.82	0.74	0.78	0.86	0.77	0.79
	Acne vs. rosacea	0.85	0.90	0.89	0.90	0.69	0.96
	Cutaneous t-cell lymphoma vs. eczema	0.74	0.84	0.81	0.88	0.63	0.90
Tschandl, P. et al.	MetaOptima	0.89			0.96		
	DAILSYLab	0.86			0.97		
	Medical Image Analysis	0.85			0.96		
Yu, Z. et al.		0.96	0.88		0.92		

SEN, sensitivity; SPE, specificity; TP, true positive; AUC, area under the receiver operating characteristic curve; PPV, positive predictive value; NPV, negative predictive value.

**Table 4:** Outcome measures of the diagnostic agreement with the gold standard with or without the use of the AI tool [10,41,44,49].

Study	Relative Difference in Diagnostic Agreement		
		Unassisted	Assisted
Dulmage, B. et al.	Agreement (%)	36	68
Jain, A. et al.	Top 1 PCP agreement (%)	48	58
	Top 3 PCP agreement (%)	58	68
	Top 1 NP agreement (%)	46	58
	Top 3 NP agreement (%)	54	66
Lucius, M. et al.	Agreement (%)	17.29	42.42
Yu, Z. et al.	AUC GP 1	0.537	0.778
	AUC GP 2	0.575	0.788

**Table 5:** Description of the training, test, and validation datasets [10,22,25,32,39,40,41,42,43,44,45,46,47,48,49].

Authors		Training Set (n Images)	Test Set (n Images)	Internal Validation	External Validation
Anderson, Jane et al.					100
Brittany Dulmage et al.		69,195	3862	3869	222
Giavina-Bianchi, M. et al.		140,446	24,000	6975	
Giavina-Bianchi, M. et al.	Dermoscopy model	21,074	2633	2635	
	Clinical model	2466	308	309	
Jain, A. et al.		64,837		14,883	
Liu, Y. et al.		64,837		14,883	
Lucius, M. et al.		8313	1702		
Muñoz-López, C. et al.		220,680	3501	17,125	340
Pangti, R. et al.		12,350	3068		5014
Phillips, M. et al.		7102			
Sangers, T. et al.					785
Soenksen, LR. et al.		20,388	6796	6796	
Thomsen, K. et al.		13,232	1657	1654	
Tschandl, P. et al.		10,015	1195		
Yu, Z. et al.		1088	136	134	

## Data Availability

The datasets generated and/or analysed during the current study are not publicly available but are available from the corresponding author on reasonable request.

## Supplementary Materials

The following supporting information can be downloaded at: https://www.mdpi.com/article/10.3390/healthcare12121192/s1, Figure S1: Quality assessment plot; Table S1: Categories and types of skin lesions and usefulness of AI tools included in every study reviewed here.

## Appendix A. Search Strategies for Bibliographic Databases

Artificial intelligence OR computer-assisted diagnosis OR clinical decision support systems OR machine learning OR neural network computer OR deep neural networks OR AI) AND (Primary health care OR Physician Primary care OR Nursing Primary care OR Primary care OR general practitioners OR Community Medicine)) AND (Skin diseases OR Dermatology), from 2012 to 2022.
