# Supplementary material for: The Use of Artificial Intelligence for Skin Disease Diagnosis in Primary Care Settings: A Systematic Review
Source: Healthcare (Basel). 2024 Jun 13;12(12):1192. doi: 10.3390/healthcare12121192 (PMC11202856; doi:10.3390/healthcare12121192)
Supplement: Supplementary file 1 [file healthcare-12-01192-s001.zip › healthcare-2973232-supplementary.pdf]

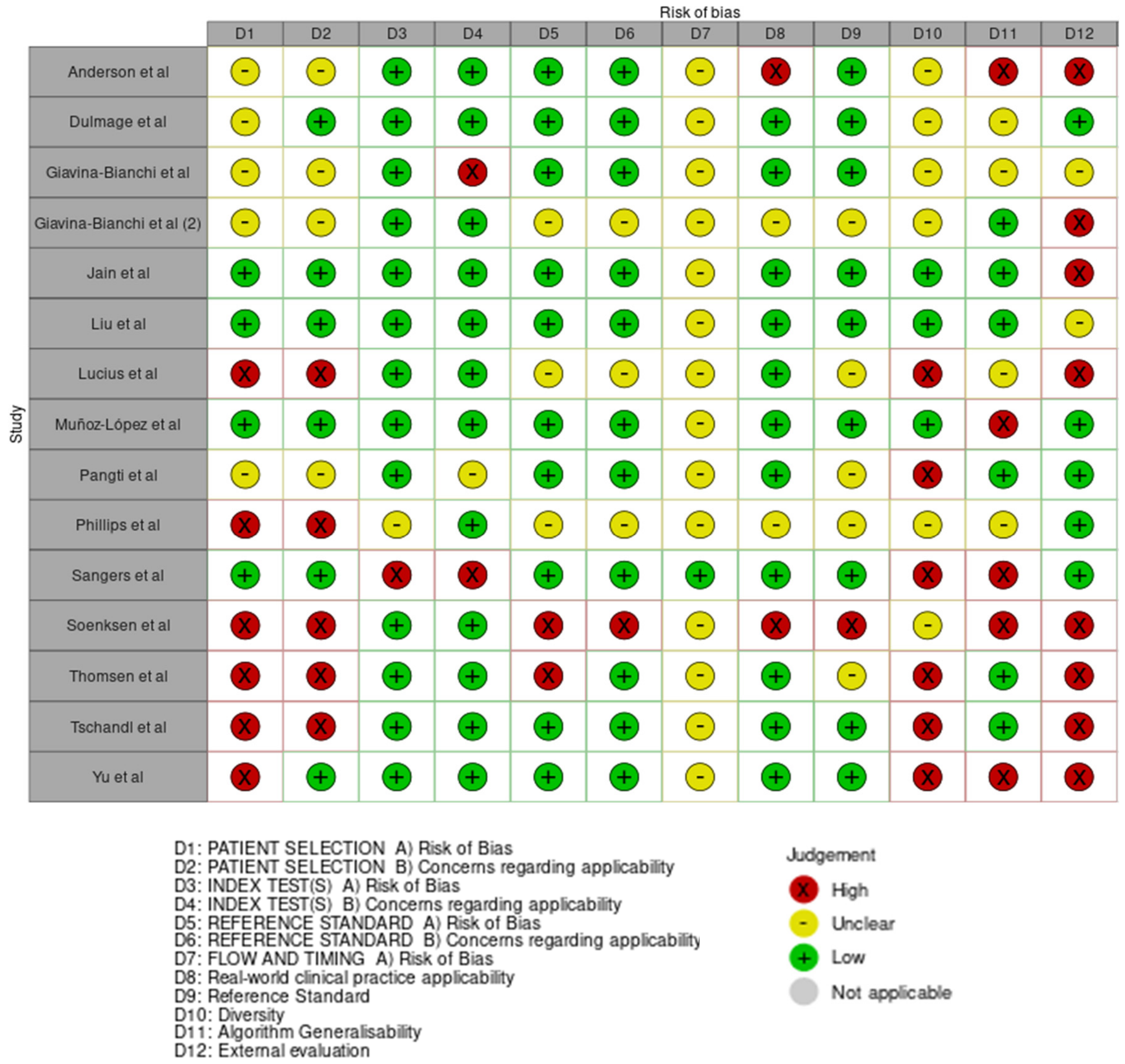

Figure S1. Quality assessment plot.

Table S1. Table of categories and types of skin lesions included in every study reviewed and the usefulness of the AI tool.

Authors	Categories of skin lesions	Types of skin lesions	Usefulness of AI
Anderson, Jane et al	Benign cutaneous tumors	Melanocytic nevus, Dermatofibroma, Seborrheic Keratosis	Triage of suspicious skin lesions
	Premalignant skin lesions	Actinic keratosis	
	Malignant cutaneous tumors	Melanoma, Basal cell carcinoma, Squamous cell carcinoma	
Brittany Dulmage et al	Primary lesion morphology		Diagnostic support tool
Giavina-Bianchi M. et al	Benign cutaneous tumors		Diagnostic support tool<br>Triage of suspicious skin lesions
	Premalignant tumoral lesions	Actinic keratosis	

	Malignant cutaneous tumors		
	Inflammatory diseases	Eczema	
	Infectious diseases	Superficial infection/Infestation	
	Others	Pigmentation disorder, Connective tissue disorder, Adverse drug reaction, Genetic cause, External cause, Bullous disease	
Giavina-Bianchi M. et al	Benign cutaneous tumors	Typical melanocytic nevus, Seborrheic keratosis, Dermatofibroma, Pyogenic granuloma, Lichenoid keratosis	Triage of suspicious skin lesions
	Premalignant skin lesions	Atypical melanocytic nevus, Actinic keratosis	
	Malignant cutaneous tumors	Melanoma, Basal cell carcinoma, Squamous cell carcinoma	
	Infectious diseases	Viral warts	
	Others	Hypertrophic scars	
Jain A et al.	Benign cutaneous tumors	Cyst, Melanocytic nevus, Seborrheic keratosis, Skin tag, Lentigo	Diagnostic support tool Triage of suspicious skin lesions
	Premalignant skin lesions	Actinic keratosis	
	Malignant cutaneous tumors	Melanoma, Basal cell carcinoma, Squamous cell carcinoma	
	Inflammatory diseases	Eczema, Psoriasis, Seborrheic dermatitis, Hidradenitis, Urticaria, Alergic contact dermatitis	
	Infectious diseases	Acne, Foliculitis, Tinea, Verruca vulgaris	
	Others	Alopecia areata, Androgenetic alopecia, Postinflammatory hyperpigmentation, Scar condition, Stasis dermatitis, Vitiligo	
Liu Y et al.	Benign cutaneous tumors	Lentigo, Melanocytic nevus, Seborrheic keratosis, Skin tag	Diagnostic support tool Triage of suspicious skin lesions
	Premalignant skin lesions	Actinic keratosis	
	Malignant cutaneous tumors	Melanoma, Basal cell carcinoma, Squamous cell carcinoma	
	Inflammatory diseases	Contact dermatitis, Eczema, Hidradenitis, Post inflammatory hyperpigmentation, Psoriasis, Seborrheic dermatitis, Urticaria	
	Infectious diseases	Acne, Folliculitis, Tinea, Tinea versicolor, Verruca vulgaris	
	Others	Alopecia areata, Androgenic alopecia, Cyst, Scar condition, Stasis dermatitis, Vitiligo	
Lucius M. et al	Benign cutaneous tumors	Melanocytic nevus, Cherry angiomas, Angiokeratomas, Pyogenic granulomas, Hemorrhages, Seborrheic keratoses, Solar lentigo, Lichen-planus-like keratosis, Dermatofibroma	Diagnostic support tool
	Premalignant skin lesions	Actinic keratosis	

	Malignant cutaneous tumors	Bowen’s disease, Basal cell carcinoma, Melanoma	
Muñoz-López C. et al	Benign cutaneous tumors	Melanocytic nevus, Seborrheic keratosis, Dermatofibroma, Epidermal cyst, Keloid, Organoid nevus, Telangiectatic granuloma	Diagnostic support tool
	Premalignant skin lesions	Actinic keratosis	
	Malignant cutaneous tumors	Basal cell carcinoma, Bowen's disease, Squamous cell carcinoma	
	Inflammatory diseases	Contact dermatitis, Eczema, Seborrheic dermatitis, Atopic dermatitis, Dyshidrotic eczema, Cheilitis, Chronic paronychia, Erythema annulare centrifugum,Lichen simplex chronicus, Morphea, Cutaneous lupus, Bullous disease, Poikiloderma, Psoriasis, Pityriasis rosea, Lichen nitidus, Pityriasis lichenoides chronica, Urticaria	
	Infectious diseases	Acne, Rosacea, Warts, Herpes zoster, Viral rash, Eczema herpeticum, Molluscum contagiosum, Tinea, Onychomycosis, Pityriasis Versicolor, Angular cheilitis, Ingrown nail, Folliculitis, Paronychia, Furuncle, Impetigo	
	Genital diseases	Condylomas, Balanitis	
	Others	Vitiligo, Androgenetic alopecia, Alopecia areata, Insect bite, Ulcers, Scar, Striae distansae, Hematoma, Erythema ab igne	
Pangti, R et al.	Benign cutaneous tumors	Melanocytic nevus, Seborrheic keratosis, Keratoacanthoma	Diagnostic support tool
	Premalignant skin lesions	Actinic keratosis	
	Malignant cutaneous tumors	Melanoma, Basal cell carcinoma, Bowen's disease, Squamous cell carcinoma	
	Inflammatory diseases	Eczema, Pityriasis rosea, Psoriasis, Urticaria, Hidradenitis suppurativa	
	Infectious diseases	Acne, Candidiasis, Herpes zoster, Impetigo and pyodermas, Molluscum contagiosum, Pityriasis versicolor, Tinea, Rosacea, Viral warts	
	Genital diseases	Anogenital warts	
	Others	Discoid lupus erythematosus, Keloids/Hypertrophic scar, Vitiligo, Alopecia, Melasma, Ichthyosis, Bullous pemphigoid, Fixed drug eruption, Lichen sclerosus, Lichen planus	
Phillips, M. et al.	Benign cutaneous tumors	Benign pigmented lesions	Triage of suspicious skin lesions

	Malignant cutaneous tumors	Melanoma	
Sangers T. et al.	Benign cutaneous tumors	Melanocytic nevus, Seborrheic keratosis, Solar lentigo,<br>Epidermal cyst, Angioma, Sebaceous gland hyperplasia	Triage of suspicious skin lesions
	Premalignant skin lesions	Dysplastic nevus, Actinic keratosis	
	Malignant cutaneous tumors	Melanoma, Basal cell carcinoma, Bowen's disease,<br>Squamous cell carcinoma, Lentigo maligna	
	Infectious diseases	Viral wart	
	Others	Hematoma, Ephelis, Excoriation, Varices	
Soenksen LR. et al.	Benign cutaneous tumors	Melanocytic nevus, Blue nevus, Clark nevus, Reed Spitz nevus, Congenital nevus, Melanosis,<br>Seborrheic keratosis, Acrochordon, Cherry angioma, Lentigo	Triage of suspicious skin lesions
	Malignant cutaneous tumors	Melanoma, Basal cell carcinoma	
Thomsen K. et al.	Malignant cutaneous tumors	Cutaneous t-cell lymphoma	Diagnostic support tool
	Inflammatory diseases	Psoriasis, Eczema	
	Infectious diseases	Acne, Rosacea	
Tschandl P. et al.	Benign cutaneous tumors	Solar lentigo, Seborrheic keratosis,<br>Lichen planus-like keratosis,<br>Dermatofibroma, melanocytic nevus,<br>Vascular lesions	Diagnostic support tool
	Premalignant skin lesions	Actinic keratoses	
	Malignant cutaneous tumors	Bowen’s disease, Basal cell carcinoma, Melanoma	
Yu Z. et al.	Inflammatory diseases	Psoriasis, Seborrheic dermatitis	Diagnostic support tool

Full List of Included Studies

1. Anderson, Jane; Tejani, Izhaar; Jarmain, Tory; Kellett, Lisa; Moy, Ronald (2022): Superiority of Artificial Intelligence in the Diagnostic Performance of Malignant Melanoma Compared to Dermatologists and Primary Care Providers. TechRxiv. Preprint. https://doi.org/10.36227/techrxiv.19657938.v1
2. Dulmage B, Tegtmeyer K, Zhang MZ, Colavincenzo M, Xu S. A Point-of-Care, Real-Time Artificial Intelligence System to Support Clinician Diagnosis of a Wide Range of Skin Diseases. J Invest Dermatol. 2021 May;141(5):1230-1235. doi:10.1016/j.jid.2020.08.027. Epub 2020 Oct 14. PMID: 33065109.
3. Giavina-Bianchi M, Cordioli E, Dos Santos AP. Accuracy of Deep Neural Network in Triaging Common Skin Diseases of Primary Care Attention. Front Med (Lausanne). 2021 Aug 26;8:670300. doi:10.3389/fmed.2021.670300. PMID: 34513863; PMCID: PMC8427035.
4. Giavina-Bianchi M, de Sousa RM, Paciello VZA, Vitor WG, Okita AL, Prôa R, Severino GLDS, Schinaid AA, Espírito Santo R, Machado BS. Implementation of artificial intelligence algorithms for melanoma screening in a primary care setting. PLoS One. 2021 Sep 22;16(9):e0257006. doi:10.1371/journal.pone.0257006. PMID: 34550970; PMCID: PMC8457457.
5. Jain A, Way D, Gupta V, Gao Y, de Oliveira Marinho G, Hartford J, Sayres R, Kanada K, Eng C, Nagpal K, DeSalvo KB, Corrado GS, Peng L, Webster DR, Dunn RC, Coz D, Huang SJ, Liu Y, Bui P, Liu Y. Development and Assessment of an Artificial Intelligence-Based Tool for Skin

Condition Diagnosis by Primary Care Physicians and Nurse Practitioners in Teledermatology Practices. JAMA Netw Open. 2021 Apr 1;4(4):e217249. doi: 10.1001/jamanetworkopen.2021.7249. PMID: 33909055; PMCID: PMC8082316.

6. Liu Y, Jain A, Eng C, Way DH, Lee K, Bui P, Kanada K, de Oliveira Marinho G, Gallegos J, Gabriele S, Gupta V, Singh N, Natarajan V, Hofmann-Wellenhof R, Corrado GS, Peng LH, Webster DR, Ai D, Huang SJ, Liu Y, Dunn RC, Coz D. A deep learning system for differential diagnosis of skin diseases. Nat Med. 2020 Jun;26(6):900-908. doi:10.1038/s41591-020-0842-3. Epub 2020 May 18. PMID: 32424212.
7. Lucius M, De All J, De All JA, Belvisi M, Radizza L, Lanfranconi M, Lorenzatti V, Galmarini CM. Deep Neural Frameworks Improve the Accuracy of General Practitioners in the Classification of Pigmented Skin Lesions. Diagnostics (Basel). 2020 Nov 18;10(11):969. doi:10.3390/diagnostics10110969. PMID: 33218060; PMCID: PMC7698907.
8. Muñoz-López C, Ramírez-Cornejo C, Marchetti MA, Han SS, Del Barrio-Díaz P, Jaque A, Uribe P, Majerson D, Curi M, Del Puerto C, Reyes-Baraona F, Meza-Romero R, Parra-Cares J, Araneda-Ortega P, Guzmán M, Millán-Apablaza R, Nuñez-Mora M, Liopyris K, Vera-Kellet C, Navarrete-Dechent C. Performance of a deep neural network in teledermatology: a single-centre prospective diagnostic study. J Eur Acad Dermatol Venereol. 2021 Feb;35(2):546-553. doi:10.1111/jdv.16979. Epub 2020 Nov 22. PMID: 33037709; PMCID: PMC8274350.
9. Pangti R, Mathur J, Chouhan V, Kumar S, Rajput L, Shah S, Gupta A, Dixit A, Dholakia D, Gupta S, Gupta S, George M, Sharma VK, Gupta S. A machine learning-based, decision support, mobile phone application for diagnosis of common dermatological diseases. J Eur Acad Dermatol Venereol. 2021 Feb;35(2):536-545. doi:10.1111/jdv.16967. Epub 2020 Nov 12. PMID: 32991767.
10. Phillips M, Greenhalgh J, Marsden H, Palamaras I. Detection of Malignant Melanoma Using Artificial Intelligence: An Observational Study of Diagnostic Accuracy. Dermatol Pract Concept. 2019 Dec 31;10(1):e2020011. doi:10.5826/dpc.1001a11. PMID: 31921498; PMCID: PMC6936633.
11. Sangers T, Reeder S, van der Vet S, Jhingoer S, Mooyaart A, Siegel DM, Nijsten T, Wakkee M. Validation of a Market-Approved Artificial Intelligence Mobile Health App for Skin Cancer Screening: A Prospective Multicenter Diagnostic Accuracy Study. Dermatology. 2022;238(4):649-656. doi:10.1159/000520474. Epub 2022 Feb 4. PMID: 35124665; PMCID: PMC9393821.
12. Soenksen LR, Kassis T, Conover ST, Marti-Fuster B, Birkenfeld JS, Tucker-Schwartz J, Naseem A, Stavert RR, Kim CC, Senna MM, Avilés-Izquierdo J, Collins JJ, Barzilay R, Gray ML. Using deep learning for dermatologist-level detection of suspicious pigmented skin lesions from wide-field images. Sci Transl Med. 2021 Feb 17;13(581):eabb3652. doi:10.1126/scitranslmed.abb3652. PMID: 33597262.
13. Thomsen K, Christensen AL, Iversen L, Lomholt HB, Winther O. Deep Learning for Diagnostic Binary Classification of Multiple-Lesion Skin Diseases. Front Med (Lausanne). 2020 Sep 22;7:574329. doi:10.3389/fmed.2020.574329. PMID: 33072786; PMCID: PMC7536339.
14. Tschandl P, Codella N, Akay BN, Argenziano G, Braun RP, Cabo H, Gutman D, Halpern A, Helba B, Hofmann-Wellenhof R, Lallas A, Lapins J, Longo C, Malvehy J, Marchetti MA, Marghoob A, Menzies S, Oakley A, Paoli J, Puig S, Rinner C, Rosendahl C, Scope A, Sinz C, Soyer HP, Thomas L, Zalaudek I, Kittler H. Comparison of the accuracy of human readers versus machine-learning algorithms for pigmented skin lesion classification: an open, web-based, international, diagnostic study. Lancet Oncol. 2019 Jul;20(7):938-947. doi: 10.1016/S1470-2045(19)30333-X. Epub 2019 Jun 12. PMID: 31201137; PMCID: PMC8237239.
15. Yu Z, Kaizhi S, Jianwen H, Guanyu Y, Yonggang W. A deep learning-based approach toward differentiating scalp psoriasis and seborrheic dermatitis from dermoscopic images. Front Med (Lausanne). 2022 Nov 3;9:965423. doi: 10.3389/fmed.2022.965423. PMID: 36405606; PMCID: PMC9669613.
